# Differences in plasma fibrin clot composition in patients with thrombotic antiphospholipid syndrome compared with venous thromboembolism

**DOI:** 10.1038/s41598-018-35034-x

**Published:** 2018-11-23

**Authors:** Aneta Stachowicz, Michal Zabczyk, Joanna Natorska, Maciej Suski, Rafał Olszanecki, Ryszard Korbut, Jacek R. Wiśniewski, Anetta Undas

**Affiliations:** 1 Chair of Pharmacology, Jagiellonian University Medical College, Krakow, Poland; 2 Biochemical Proteomics Group, Department of Proteomics and Signal Transduction, Max Planck Institute of Biochemistry, Martinsried, Germany; 3 Institute of Cardiology, Jagiellonian University Medical College, Krakow, Poland; 4 Krakow Center for Medical Research and Technology, John Paul II Hospital, Krakow, Poland

## Abstract

The prothrombotic fibrin clot phenotype has been reported in patients with thrombotic antiphospholipid syndrome (APS) and venous thromboembolism (VTE). Protein composition of plasma fibrin clots in APS has not been studied. We evaluated 23 patients with thrombotic APS, 19 with VTE alone, and 20 well-matched controls. A proteomic analysis of fibrin clots generated from citrated plasma was based on liquid chromatography-mass spectrometry. Plasma levels of thrombospondin-1 (TSP1), apolipoprotein(a), A-I, and B-100, complement components (C)3a, C5b-C9, histidine-rich glycoprotein (HRG), and prothrombin were evaluated using immunoenzymatic tests. In plasma fibrin clots of APS patients, compared with VTE subjects and controls, we identified decreased amounts of (pro)thrombin, antithrombin-III, apolipoprotein A-I, and HRG with no differences in plasma levels of antithrombin, prothrombin, along with lower plasma HRG and apolipoprotein A-I. In APS patients, plasma HRG positively correlated with amounts of clot-bound HRG, while apolipoprotein A-I was inversely associated with clot-bound levels of this protein. The most predominant proteins within the clots of APS patients were bone marrow proteoglycan, C5-C9, immunoglobulins, apolipoprotein B-100, platelet-derived proteins, and TSP1. Our study is the first to demonstrate differences in the protein composition of fibrin clots generated from plasma of thrombotic APS patients versus those with VTE alone.

## Introduction

Antiphospholipid syndrome (APS) is an autoimmune disease characterized by a hypercoagulable state associated with vascular thrombosis and/or pregnancy morbidity in the presence of circulating antiphospholipid antibodies (aPL) including immunoglobulin (Ig)G and IgM antibodies to β-2Glycoprotein I (β2GpI) and anticardiolipin antibodies (aCL)^1^. aPL are observed in up to 10% of patients with deep vein thrombosis (DVT) with or without pulmonary embolism (PE)^2^. Multiple prothrombotic mechanisms associated with the presence of aPL have been demonstrated including enhanced blood coagulation and impaired fibrinolysis^3^. Looking for new prothrombotic mechanisms underlying thrombotic APS, in 2014 we demonstrated in APS patients the so-called prothrombotic clot phenotype involving faster formation of denser fiber networks with the subsequent lower clot permeability and lysability^4^, which has been confirmed by Vikerfors *et al*.^5^. Recently, it has been shown that β2GpI at physiological conditions is bound to fibrinogen^6^, which may contribute to prothrombotic effects of anti-β2GpI antibodies in APS patients.

*In vitro* studies demonstrated that fibrin binds in a covalent and non-covalent manner a large variety of proteins^7,8^. The first report on proteomics of fibrin clots generated from plasma obtained from healthy volunteers was published by Talens *et al*.^9^ in 2012. Using 2D gel electrophoresis, mass spectrometry, and Western blot analysis they showed 18 non-covalently bound proteins, which were mostly associated with blood coagulation, protease inhibition, and metabolism of high-density lipoproteins. Proteomics of fibrin clots generated from plasma of patients with acute myocardial infarction (AMI) using shotgun method (2DLC-MS/MS) identified 62 proteins belonging to several distinct functional clusters (e.g. cholesterol transporter activity, immunoglobulin binding and peptidase regulatory activity)^7^. Our previous study revealed 476 proteins repeatedly identified in the plasma fibrin clots from 4 patients with VTE including extracellular vesicle-derived proteins, lipoproteins, fibrinolysis inhibitors, and proteins involved in immune responses^6^. The main proteins identified within clot generated from plasma of VTE patients were fibrinogen, along with fibronectin, alpha-2-antiplasmin, alpha-2-macroglobulin, and factor (F)XIII, while others, such as FVIII, FXII, von Willebrand factor (vWF), and plasminogen were present at very low concentrations^6^.

To our knowledge, there have been no reports on the composition of plasma fibrin clots in patients with thrombotic APS. Employing label-free quantitative proteomics we aimed to investigate the protein composition of plasma clots prepared *ex vivo* from patients with APS-associated VTE versus those with VTE unrelated to APS and age- and sex-matched healthy controls.

## Material and Methods

### Patients

We investigated 42 white patients with a history of VTE experienced 7 months or more prior to enrolment, including 23 patients with APS referred for thrombophilia screening or further laboratory work-up. Twenty age- and sex-matched healthy subjects were served as controls. The exclusion criteria were current oral anticoagulation, signs of acute infection, known malignancy, end-stage renal insufficiency, and liver injury. The diagnosis of APS was established based on the modified classification criteria^10^. The diagnosis of PE was based on the presence of typical symptoms and positive results of high resolution spiral computed tomography. The diagnosis of DVT was established by a positive finding of color duplex sonography of lower extremity veins. Ischemic stroke, myocardial infarction and other comorbidities were diagnosed as described^4^. The Jagiellonian University Bioethics Committee approved of the study and the informed consent was obtained from all the participants in accordance with the Declaration of Helsinki.

### Laboratory investigations

Blood samples were drawn from an antecubital vein with minimal stasis using atraumatic venipuncture at 8 to 10 AM. At the time of blood drawing none of the subjects was taking vitamin K antagonists (VKA). Patients on VKA were switched to enoxaparin for two weeks and blood was collected 24 h since the last injection when International Normalized Ratio (INR) was < 1.2 and anti-factor Xa plasma activity was < 0.2 IU/ml. Blood samples (vol/vol, 9:1 of 3.2% trisodium citrate) were spun at 2000x g for 10 minutes, and the supernatants were aliquoted and stored at −80 °C for fibrin analysis. Blood cell count, lipid profiles, glucose, creatinine, activated partial thromboplastin time (aPTT) and INR were assayed by routine laboratory techniques. Fibrinogen was determined using the Clauss method. High-sensitivity C-reactive protein (CRP) was measured by nephelometry (Siemens, Marburg, Germany). In VTE patients immunoenzymatic assays were performed to assess plasma/serum levels of histidine-rich glycoprotein (HRG), thrombospondin 1 (TSP1), prothrombin (all from Biosource, Camarillo, CA, USA), complement C3a, C5a, C5b-C9 (all Quidel, San Diego, CA, USA), apolipoprotein A-I (apo A-I), apolipoprotein B-100 (apo B-100) (both R&D Systems, Abingdon, UK), and lipoprotein(a) (Lp(a), DRG International Inc., Springfield, NJ, USA).

All patients were screened for thrombophilia, including the factor V Leiden mutation (FVL), prothrombin G20210A, or deficiency of natural anticoagulants. Inherited thrombophilia was defined as the presence of either of the two mutations or a deficiency of one of the three coagulation inhibitors. Genotypes of FV Leiden (rs6025) and prothrombin G20210A (rs1799963) polymorphisms were ascertained by the allelic discrimination test using the TaqMan Genotyping assay on the ABI PRISM 7900HT Fast Real-Time PCR System (Life Technologies Co., Carlsbad, CA, USA).

Estimation of lupus anticoagulant (LA) was performed using a clot-based assay as recommended. Anticardiolipin and anti-β2GP-I antibodies were determined by immunoenzymatic assays (INOVA Diagnostics, San Diego, USA). Reference ranges for IgG were up to 15 IgG phospholipid units (GPL) and 8 standard IgG β2Gp units (SGU), respectively, and for IgM up to 17 IgM phospholipid units (MPL) and 10 standard IgM β2Gp units (SMU), respectively. All positive cases we reevaluated after 12–16 weeks. A single antibody positivity we defined as the presence of: immunoglobulin M and/or IgG aCL, anti-β2GpI or LA alone, a double positivity as: the presence of IgM and/or IgG for aCL + anti-β2GpI or aCL + LA or anti-β2GpI + LA, and a triple positivity as positive IgM and/or IgG aCL + anti-β2GpI + LA^11^. Systemic lupus erythromatosus (SLE) was diagnosed based on the American College of Rheumatology classification criteria^12^.

All measurements were performed by technicians blinded to the origin of the samples.

#### Plasma clot preparation and lysis

A fibrin clot was prepared using an assay recommended by the Factor XIII and Fibrinogen Subcommittee of the Scientific Standardisation Committee of the International Society on Thrombosis and Haemostasis^13^. Briefly, to 100 μL of citrate plasma was added 20 mmol/L calcium chloride and 1 U/mL human thrombin (Merck, Kenilworth, NJ, USA). This mixture was placed into plastic tubes, which were put into a wet chamber. After 120 minutes of incubation tubes were connected to a reservoir of a buffer (0.05 mol/L Tris HCl, 0.1 mol/L NaCl, pH 7.5). Clots were vigorously rinsed. Then the clots were immediately frozen at −80 °C. Clots were lysed in a buffer containing 0.1 M Tris-HCl, pH 8.0, 1% sodium dodecyl sulfate and 50 mM dithiothreitol (all reagents, Sigma Aldrich, St. Louis, MO, USA) at 96 °C for 10 min.

#### Label-free quantitative proteomics

Label-free quantitative proteomics of fibrin clots prepared *ex vivo* from citrated plasma of all subjects was performed as previously described^6^. Briefly, a total protein concentration in lysates and the peptide contents in the digests were assayed using a tryptophan fluorescence based WF-assay^6,14^. The multiple enzyme digestion filter-aided sample preparation (MED-FASP) method^15,16^ with three enzymes: endoproteinase LysC, trypsin and chymotrypsin was used to digest proteins. Analysis of peptide mixtures was performed using a QExactive HF mass spectrometer (Thermo-Fisher Scientific, Waltham, MA, USA) operated in the data-dependent mode with HCD fragmentation. The mass spectrometry data were deposited to the ProteomeXchange Consortium via the PRIDE partner repository^17^ with the dataset identifier: PXD008434. The spectra were searched using Andromeda search engine built-in MaxQuant software. The maximum false peptide and protein discovery rate was specified as 0.01. Relative protein quantification was performed using the MaxQuant label-free algorithm (MaxLFQ)^18^. Statistical analysis of proteomic data was conducted by Perseus software^19^.

### Statistical analysis

Variables are presented as numbers (percentages), mean ± standard deviation (SD) or median and interquartile range (IQR) as appropriate. Normal distribution was assessed by Shapiro-Wilk test. Equality of variances was assessed using the Levene’s test. Differences between groups were compared using the Student’s or the Welch’s t-test depending on the equality of variances for normally distributed variables. The Mann-Whitney U-test was used for non-normally distributed continuous variables. Categorical variables were compared by the Fisher’s exact test. LFQ intensities calculated by MaxQuant label-free algorithm for each protein present in the control, VTE, and APS groups were compared using one-way ANOVA and p-values were adjusted with the Permutation-based False Discovery Rate (FDR) correction. A two-sided p-value < 0.05 was considered statistically significant.

## Results

### Patient characteristics

The three study groups are presented in Table 1. VTE patients experienced the event at a median of 20 (IQR 12–34, minimum 7) months prior to enrolment. Among 23 APS patients, 5 had a single, 3 double, and 15 triple antibody positive APS. Detectable values of anti-Xa activity were found only in a subset of APS patients (n = 13, 56.5%; median, 0.12 [0.07–0.15] IU/ml). There were no differences between APS and VTE patients with regard to the medication used (Table 1). APS patients had 13.7% and 25% higher fibrinogen levels than VTE patients and controls, respectively. We observed lower low-density lipoprotein cholesterol (LDL-C) levels in the APS group compared with VTE patients and controls, while higher triglycerides (TG) were found in APS patients. Factor V Leiden and/or prothrombin G20210A mutations were detected in 2 (10.5%) VTE patients, while 1 subject heterozygous for FVL mutation was observed in the APS and control groups. No deficiencies of natural anticoagulants were found. Compared with VTE subjects, APS patients had higher plasma levels of TSP1 (104 [98–110] vs. 99 [86–103] ng/ml, p = 0.024), while lower apo A-I (102 [98–112] vs. 112 [101–127] mg/dl, p = 0.04), and HRG (40 [36–45] vs. 47 [39–55] µg/ml, p = 0.0067). Moreover, APS patients were characterized by elevated plasma levels of complement components C3a, C5a, and C5b-C9 (all p < 0.0001). There were no differences between VTE and APS patients in plasma levels of apo B-100 (97 [76–103] vs. 83 [72–105] mg/dl, p = 0.63), Lp(a) (10.7 [5.9–32] vs. 17.6 [9.8–30.5] mg/dl, p = 0.21), and prothrombin (123.5 [106–146] vs. 130 [119–146] µg/ml, p = 0.37).

**Table 1:** Characteristics of patients with thrombotic APS, VTE, and healthy controls.

Variable	A. Thrombotic APS (n = 23)	B. VTE (n = 19)	C. Healthy controls (n = 20)	P-value A vs. B	P-value A vs. C	P-value B vs. C
Age, years	38 (30–53)	42 (35–47)	40 (31–49)	0.71	0.98	0.65
Male, n (%)	3 (13)	3 (16)	5 (25)	0.99	0.44	0.70
Body mass index, kg/m^2^	27.0 (23.8–29.7)	23.4 (21.6–28.7)	23.7 (21.1–25.7)	0.20	0.02	0.84
**Clinical characteristics**						
Current smoking, n (%)	5 (22)	2 (11)	5 (25)	0.44	0.54	0.99
Family history of VTE, n (%)	5 (22)	5 (27)	0 (0)	0.72	0.051	0.017
DVT alone, n (%)	7 (30)	8 (42)	0 (0)	0.74	0.01	0.003
PE, n (%)	16 (70)	11 (58)	0 (0)	0.74	<0.001	<0.001
Ischemic stroke, n (%)	9 (39)	0 (0)	0 (0)	0.003	0.002	0.99
Myocardial infarction, n (%)	2 (9)	0 (0)	0 (0)	0.50	0.49	0.99
Diabetes mellitus, n (%)	1 (4)	3 (16)	0 (0)	0.22	1.00	0.10
Arterial hypertension, n (%)	9 (39)	3 (16)	0 (0)	0.17	0.002	0.10
Lupus anticoagulant, n (%)	15 (65)	0 (0)	0 (0)	<0.001	<0.001	0.99
Systemic lupus erythromatosus, n (%)	12 (52)	0 (0)	0 (0)	<0.001	<0.001	0.99
**Medication**						
Prior anticoagulation, n (%)	19 (83)	13 (72)	0 (0)	0.47	<0.001	<0.001
Aspirin, n (%)	7 (30)	3 (16)	0 (0)	0.27	0.023	0.021
ACEI, n (%)	5 (22)	2 (11)	0 (0)	0.33	0.051	0.45
Statin, n (%)	1 (4)	0 (0)	0 (0)	0.92	0.94	0.99
**Laboratory investigations**						
Fibrinogen, g/L	3.40 (3.10–4.00)	2.99 (2.58–3.07)	2.72 (2.34–3.05)	0.029	0.001	0.23
D-dimer, ng/mL	564 (276–870)	344 (253–465)	287 (203–465)	0.26	0.08	0.29
Glucose, mmol/L	4.7 (4.4–5.0)	5.2 (4.9–5.3)	4.9 (4.6–5.1)	0.004	0.27	0.032
Creatinine, µmol/L	71 (62–87)	72 (61–80)	68 (60–85)	0.60	0.59	0.97
Total cholesterol, mmol/L	5.1 (4.4–5.6)	4.9 (4.6–5.7)	5.2 (4.6–5.9)	0.96	0.42	0.68
LDL-C, mmol/L	1.5 (1.1–2.9)	3.1 (2.6–4.3)	3.1 (2.7–3.6)	0.001	<0.001	0.84
HDL-C, mmol/L	1.8 (1.4–2.7)	1.9 (1.3–2.1)	1.8 (1.6–2.0)	0.45	0.76	0.91
Triglycerides, mmol/L	1.80 (1.08–2.42)	0.84 (0.71–1.19)	0.97 (0.7–1.20)	<0.001	<0.001	0.59
hsCRP, mg/L	1.86 (1.10–4.10)	0.89 (0.53–2.27)	0.84 (0.59–2.11)	0.07	0.058	0.92
Antithrombin, %	110 (103–114)	101 (96–112)	104 (94–112)	0.12	0.037	0.69

Values are median (first-third quartile). Abbreviations: APS, antiphospholipid syndrome; DVT, deep vein thrombosis; HDL-C, high density lipoprotein cholesterol; hsCRP, high sensitivity C-reactive protein; LDL-C, low density lipoprotein cholesterol; PE, pulmonary embolism; VTE, venous thromboembolism.

### Label-free quantitative proteomics of fibrin clots

**Figure 1:**
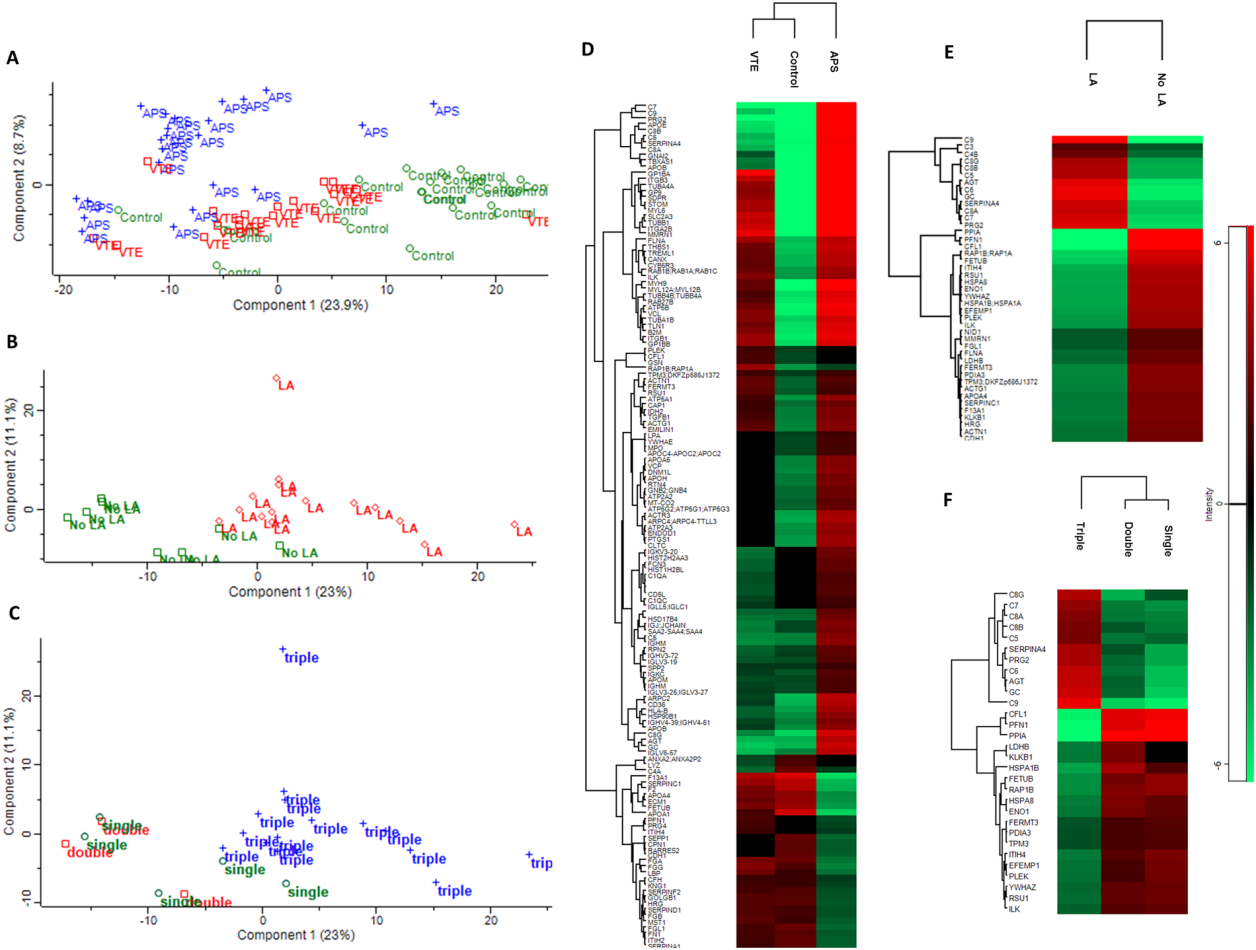
Principal component analysis of the proteomic data of fibrin clots prepared from plasma of patients with APS (n = 23), VTE (n = 19) and healthy controls (n = 20) (**A**) and from patients with APS in the presence or absence of lupus anticoagulant (LA vs. No LA) (B) as well as from patients with triple-, double- and single-positive APS (**C**) in a 2D graph of principal component 1 and component 2. Heat map presentation of a hierarchical cluster of significantly changed (p < 0.05) proteins in fibrin clots from patients with APS, VTE and healthy control (**D**) and from patients with APS in the presence or absence of lupus anticoagulant (LA vs. No LA) (E) as well as from patients with triple-, double- and single-positive APS (**F**). The green and red colors represent low and high levels, respectively.

**Figure 2:**
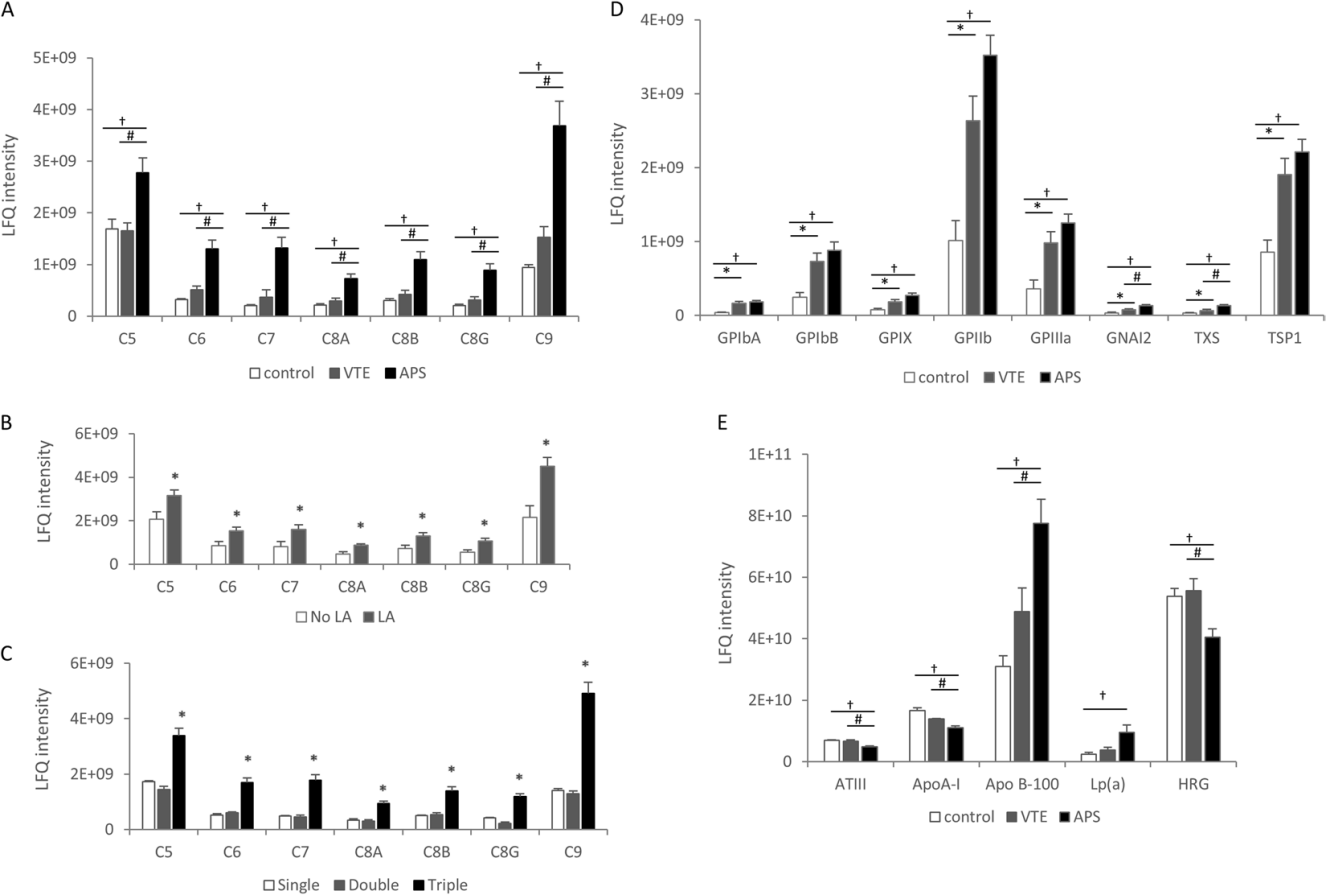
Relative amounts of C5-C9 complement components in fibrin clots from plasma of patients with APS (n = 23), VTE (n = 19) and healthy controls (n = 20) (**A**) and from patients with APS in the presence or absence of lupus anticoagulant (LA vs. No LA) (**B**) as well as from patients with triple-, double- and single-positive APS (**C**). Relative amounts of proteins participated in platelets activation and aggregation (**D**) as well as proteins related to antithrombotic and prothrombotic effects (**E**) in fibrin clots from plasma of patients with APS, VTE and healthy controls. Abbreviations: LFQ, label free quantification. *p < 0.05 for VTE vs. healthy controls or LA vs. No LA and triple-positive APS vs. double- and single-positive APS; ^#^p < 0.05 for APS vs. VTE; ^†^p < 0.05 for APS vs. healthy controls.

To identify common patterns in the obtained proteomic dataset we used principal component analysis (PCA), which exposes biological diversity of human samples and indicates that samples belonging to the same group (e.g. healthy control, VTE, or APS) display common similar patterns of changes in protein incorporation. Indeed, PCA revealed similar patterns among samples in the same group (Fig. 1A–C). Different contents of proteins were found in plasma fibrin clots in the studied groups, namely 111 proteins in the APS group versus healthy controls, 48 proteins in the VTE group as compared to healthy controls and 63 proteins in the APS group as compared to the VTE group (Supplemental Table 1, Fig. 1D). Analysis of the protein content in plasma clots from patients with APS with the presence or absence of LA showed that 43 proteins were associated with the presence of LA, including bone marrow proteoglycan (PRG2), C3-C9 or HRG (Supplemental Table 1, Fig. 1E). Interestingly, triple-positive APS patients significantly differed in the content of 30 proteins in plasma fibrin clots as compared to double- and single-positive APS patients, including PRG2 or C4-C9 (Supplemental Table 1, Fig. 1F). There were no similar differences with regard to concomitant SLE, PE, or DVT. In plasma fibrin clots of thrombotic APS patients compared with the remaining VTE subjects or controls we observed decreased content of antithrombin-III (ATIII), prothrombin (F2), FXIII alpha chain, apo A-I, and HRG (all p < 0.05) in clots (Supplemental Table 1). On the contrary, variable fragments of complement components (C) C5, C6, C7, C8A, C8B, C8G, C9, together with PRG2 were highly abundant in clots generated from plasma of APS patients compared with VTE patients and healthy controls (Fig. 2A, Supplemental Table 1). Separate analysis for the APS group showed that in clots of subjects with positive LA or triple-positive APS complement components C5-C9, along with PRG2 were increased when compared with negative LA as well as single- and double-positive APS patients (Fig. 2B and C). We have observed no differences in amounts of above mentioned proteins when analyzed a potential influence of residual heparin activity (data not shown). In clots generated from plasma of both APS and VTE patients the proteins involved in platelet adhesion, activation, and aggregation, like fermitin family homolog 3, multimerin-1, TREM-like transcript-1, integrin alpha-IIb (GPIIb), integrin beta-1 (GPI), integrin beta-3 (GPIII), integrin-linked protein kinase, talin-1, vinculin, filamin A, platelet glycoprotein IX (GPIX), platelet glycoprotein Ib alpha and beta chain (GPIbA, GPIbB), and TSP1 were overrepresented as compared to healthy controls (Fig. 2D, Supplemental Table 1). Moreover, in the APS group compared to VTE patients or healthy controls, we observed within the clots increased amounts of proteins involved in platelet activation and aggregation, such as platelet glycoprotein 4 (CD36), adenylate cyclase-inhibiting G alpha protein (GNAI2), and thromboxane-A synthase (TXS) (Fig. 2D, Supplemental Table 1). Increased amounts of apo B-100, Lp(a), and various immunoglobulin chains were also observed within the clots generated from plasma of APS patients (Fig. 2E, Supplemental Table 1). In APS patients compared to controls, we also found 2.37-fold increased content of neutrophil-derived MPO in plasma clots (Supplemental Table 1). Of note, we found higher content of histones H2A and H2B in plasma fibrin clots of APS patients as compared to those with VTE without APS (Supplemental Table 1). Interestingly, we detected increased amounts of clot-bound β2GpI in APS patients as compared to controls with no difference between the APS and VTE groups (Supplemental Table 1).

In APS, but not in VTE patients plasma C5b-C9 correlated with clot-bound C5-C9 amounts (r = 0.46, p = 0.031) and plasma HRG was associated with the clot-bound HRG (r = 0.34, p = 0.028), while negative associations were observed between plasma and clot-bound apo A-I (r = −0.64, p = 0.0011) and apo B-100 (r = −0.39, p = 0.048).

## Discussion

Recently, we have shown that a multiple enzyme digestion filter aided sample preparation (MED-FASP) method combined with a label-free quantification on Q Exactive HF is an excellent proteomic approach for the analysis of fibrin clots prepared *ex vivo* from citrate plasma of 4 VTE patients^6^. Here, we have applied the above workflow to assess the protein composition of fibrin clots prepared from plasma of patients with thrombotic APS. To our knowledge this study is the first to show differences in the protein composition of plasma clots in patients with thrombotic APS compared with VTE patients without APS and matched healthy controls. This study provides insights into specific changes within fibrin clot composition in thrombotic APS, suggesting that a number of proteins, including those beyond blood coagulation components, such as inflammatory proteins, might be involved in thrombus formation and affect its properties. In plasma clots from APS patients, we identified proteins of potential relevance which were not previously demonstrated, i.e. high amounts of PRG2, C4-C9, platelet glycoproteins or TSP1 as well as low amounts of ATIII or prothrombin. In our opinion, a detailed proteomic analysis of fibrin clots in APS as well as other prothrombotic disorders might increase our knowledge about the final stage of blood coagulation and its modulation and facilitate development of new fibrin related therapies. We detected increased amounts of clot-bound complement components, immunomodulatory, and platelet-derived proteins mediating complement activation, which supports the role of complement activation and inflammation in the pathophysiology of thrombotic APS. It is known that antibodies directed against β2GpI and aCL lead to complement activation, which are associated with clinical manifestations of APS^20^. In clots generated from plasma of APS patients we found elevated amounts of proinflammatory proteins potentially associated with immunothrombosis, such as PRG2, MPO, or histones. These findings appears to provide additional evidence for enhanced release of neutrophil extracellular traps (NETs) in thrombotic APS, as shown by Yalavarthi *et al*. who reported that IgG isolated from APS patients, involving IgG anti-β2GpI, interacts with neutrophils to stimulate NETosis^21^. The present study not only supports involvement of NETs in a persistent prothrombotic state in APS patients who experienced VTE in the past, but also demonstrates the presence of NETs formation-related proteins in fibrin clots in this disease. Interestingly, in APS patients we observed highly increased amounts of clot-bound PRG2, a major protein of eosinophil granules, which is involved in the release of histamine from mast cells and basophils, and activates both neutrophils and macrophages^22^. Increased PRG2 amounts in clots may suggest an involvement of eosinophil-derived proteins in immunothrombosis and prothrombotic clot properties as previously reported in patients with eosinophilia^23^ or could be in part related to extracellular traps release. However, further studies are needed to explore a specific role of PRG2 in fibrin clot properties among subjects with normal eosinophil count, including those with APS.

**Figure 3:**
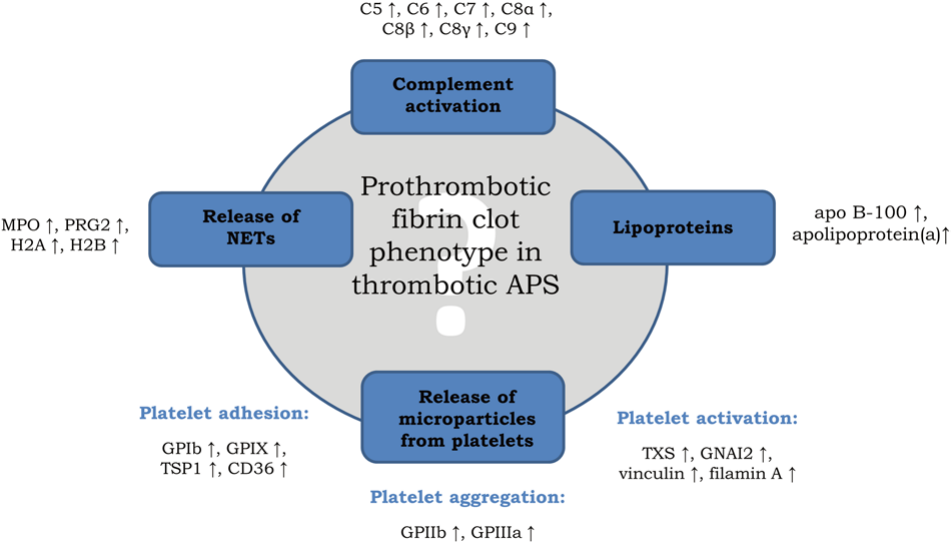
Key processes in which different amounts of clot-bound proteins, such as complement components, lipoproteins, platelet-derived proteins, and components of neutrophil extracellular traps might be associated with prothrombotic clot phenotype in APS patients. Abbreviations: C1-C9, complement components 1–9; MPO, myeloperoxidase; PRG2, bone marrow proteoglycan; H2A/B, histone 2A or B; apo B-100, apolipoprotein B-100; GPI/III/IX, platelet glycoproteins I, III, IX; TSP1, thrombospondin-1; CD36, platelet glycoprotein 4; TXS, thromboxane synthase; GNAI2, adenylate cyclase-inhibiting G alpha protein. ↑ denotes higher amounts of particular clot-bound proteins.

Previously, complement C3 within the plasma clot has been described as a novel clot component, suggesting its role in cardiovascular thromboembolic events^24^. Clinical studies have shown an involvement of the complement system in thrombotic APS. APS patients exhibit higher C3a, C4a, and C5a plasma levels than healthy controls, and their role in thrombotic disorders has been suggested^25^. Our study has shown increased plasma levels of C3a, C5a, and C5b-C9 in APS patients, which agrees with previous observations^20,26^. However, C5-9, but not C3 or other components were increased within plasma clots of APS patients compared with VTE. C5-C9 can activate platelets and increase their procoagulant activity^27^. In APS patients, complement activation within the clots can amplify coagulation and inhibit fibrinolysis^28^, promoting unfavorable clot properties, including its reduced permeability and susceptibility to lysis^4^ which might contribute to increased risk of thromboembolic events in this disease^29^. The study by Arachchillage *et al*.^25^, which has shown that complement activation was decreased in patients with thrombotic APS treated with a direct oral anticoagulant, rivaroxaban might support our hypothesis. Taken together, our findings supports the view that immune mechanisms are of key functional importance as modulators of thrombosis in APS^30^. Interestingly, the amount of complement components, together with C3, was increased in the fibrin clots from patients with APS and positive LA, which is a potent predictor of thrombosis stronger than aCL antibodies^31^. The generated C5a together with anti-β2GpI-1 antibodies in complexes with β2GpI^32^ contribute to the prothrombotic tendency in APS patients by the induction of platelet activation and thromboxane A2 (TXA2) synthesis via GPIb-IX-V complex^33,34^. Indeed, in the fibrin clots from patients with APS, as compared to healthy controls, we found increased amounts of β2GpI, along with TXS and platelet GPIbA, GPIbB, and GPIX (Fig. 3).

Our proteomic approach also pointed to the higher content of proteins participating in platelet adhesion (TSP1, CD36, fermitin family homolog 3, multimerin-1)^35,36^, activation (GNAI2, TREM-like transcript-1, integrin-linked protein kinase, talin-1, vinculin, filamin A)^37–39^ and aggregation (GPIIb, GPIIIa) in plasma clots from both APS and VTE patients as compared to healthy controls (Fig. 3). Interestingly, the content of TXS and GNAI2 in the fibrin clots was more than two times higher in APS patients than in the VTE group. TXS is an enzyme responsible for the production of TXA2, that increases platelet activation and aggregation, while GNAI2 not only mediates platelet activation, but also is involved in cerebral and myocardial ischemia/reperfusion injury *in vivo*^40^. Given a significance of aspirin in APS therapy^41^ a role of platelets in a prothrombotic state in APS requires further investigation.

We have confirmed by the bioinformatic analysis that fibrin clots from patients with VTE were enriched in extracellular vesicles, such as platelet microparticles^6^. Moreover, in APS patients compared to VTE patients and controls we found increased amounts of platelet microparticle-bound integrin GPIIb/GPIIIa supporting previous reports based on data in circulating blood^42^ and it is well known that microparticles increase risk of VTE and arterial thrombosis^43^. The present study provides additional evidence suggesting involvement of platelet particles in prothrombotic alterations in APS.

We also noticed that apo B-100 and Lp(a) amounts were elevated in the fibrin clots generated from plasma of patients with APS compared to VTE and healthy subjects. The protein component of human Lp(a) consists primarily of two apolipoproteins, apo(a) and apo B-100, linked through a cystine disulfide(s), which have been reported to represent independent risk factors for atherosclerosis and VTE^44^. Moreover, apo B-100 in complex with aCL antibodies and β2GpI exhibits its prothrombotic properties by inducing tissue factor expression on monocytes^45^. Lp(a) levels have been associated with key fibrin clot properties in healthy individuals and patients with a history of myocardial infarction^44^. In patients with residual vein obstruction prothrombotic plasma fibrin clot phenotype was related to elevated Lp(a)^46^. Thus, it is likely that elevated amounts of major components of Lp(a) bound to fibrin could modulate clot structure and function contributing to immunothrombosis in APS patients.

Our study showed reduced amounts of HRG in fibrin clots generated from plasma of APS patients, which is a novel intriguing finding. HRG plays a role in angiogenesis, apoptosis, immune regulation, and hemostasis by displaying anticoagulant and antifibrinolytic properties as shown *in vitro* via multiple mechanisms, including binding DNA and RNA and the activation of intrinsic coagulation pathway^47^. In knockout mice lacking HRG exhibited a procoagulant phenotype and developed accelerated arterial thrombosis^48^. HRG circulates in plasma bound to fibrinogen and this complex remains intact when fibrinogen is converted to fibrin^49^. HRG deficiency in humans resulted in shortened plasma clotting times and has been associated with a thrombotic phenotype^49^. Low amounts of HRG in clots and plasma of patients with thrombotic APS suggest a previously unknown role of this protein, which deserves further investigation.

An intriguing issue is the lack of differences in i.e. vWF or fibrinolysis activators and inhibitors between the groups. However, is it likely that within a mature clot, some proteins are present at low concentrations due to low plasma amounts. Our data suggests that protein incorporation during clot formation is not a passive mechanism reflecting plasma concentration of a given protein and even small changes in clot composition might significantly alter clot biophysical characteristics and its degradation. Compact, less permeable, and poorly lysable fibrin networks are typical features, which characterize a prothrombotic fibrin clot phenotype^50^. Fibrin clot permeability (K_s_), reflected by the Darcy constant, is a key measure of plasma clot structure^51^. Plasma clots with reduced K_s_ have been described in patients with VTE^52^ and APS^4,5^. Interestingly, clots prepared from plasma of patients with thrombotic APS are more compact than those from VTE alone^4^. Intergroup differences in fibrin clot proteomics observed in the current study cannot be explained by the differences in clot permeability despite the fact that physical clot features may affect the results of proteomic analysis. Although clots prepared from plasma obtained from patients with thrombotic APS are more compact than those from VTE patients^4^, we have shown significant differences not only in protein quantity but primarily in its composition, including larger amounts of disease-specific proteins such as complement components in APS. However, the influence of K_s_ on clot protein composition needs further investigation.

This study has several limitations. First, the sample size was relatively limited, however representative for the APS patients and it is unlikely that the differences reported here result from a significant recruitment bias. Second, it was a cross-sectional study and our analysis was based on a determination of each variable at a single time point, therefore some changes in fibrin clots composition over time could be observed. The current study should be perceived as hypothesis-generating, which requires further investigation on a larger cohort of APS patients to be validated. Third, due to the fact that most of the proteins are bound to fibrinogen and plasma fibrinogen concentrations differed between groups, an adjustment for fibrinogen ratio has been made and clot-bound protein fold change above 1.25 has been considered significant. Additional experiments using clots with standardized fibrinogen added to fibrinogen-depleted plasma should be performed to confirm our observations. Fourth, it has been established that the presence of fibrinogen gamma’ chain unfavorably affects the fibrin clot structure^53^ and might influence clot proteomics. However, investigation of the fibrinogen gamma’ was beyond the scope of the current study. Finally, potential influence of heparin activity on fibrin clot composition should be considered. To minimize the impact of residual activity of enoxaparin on fibrin clot formation and structure we have used exogenous thrombin (1U/ml), instead of tissue factor to initiate fibrinogen polymerization independently of thrombin generation^54^.

In conclusion, our study is the first to show the comprehensive analysis of plasma fibrin clot components and differences in protein composition between patients with thrombotic APS, compared to those with VTE and healthy controls. Our findings suggest the role of the upregulated complement components and platelet proteins as well as downregulated antithrombotic proteins, especially HRG, in patients with thrombotic APS. Thus, our clot proteomic approach could be useful to identify plasma proteins with potential clinical utility as biomarkers in thrombotic diseases. Further investigations are required to elucidate the impact of several identified proteins on plasma fibrin clot properties in thrombosis *in vivo*.

The datasets generated and analyzed during the current study are available in the PRIDE repository, PXD008434.

## Electronic supplementary material

Dataset 1
