# Supplementary material for: Differences in plasma fibrin clot composition in patients with thrombotic antiphospholipid syndrome compared with venous thromboembolism
Source: Sci Rep. 2018 Nov 23;8:17301. doi: 10.1038/s41598-018-35034-x (PMC6251889; doi:10.1038/s41598-018-35034-x)
Supplement: Supplementary file 1 — Dataset 1 [file 41598_2018_35034_MOESM1_ESM.docx]

**Differences in plasma fibrin clot composition in patients with thrombotic antiphospholipid syndrome compared with venous thromboembolism**

#Aneta Stachowicz^1,2^, #Michal Zabczyk^3^, #Joanna Natorska^3,4^, Maciej Suski^1^, Rafal Olszanecki^1^, Ryszard Korbut^1^, #Jacek R Wiśniewski^2^, and *#Anetta Undas^3,4^

#Authors equally contributed, *Corresponding author

^1^Chair of Pharmacology, Jagiellonian University Medical College, Krakow, Poland; ^2^Biochemical Proteomics Group, Department of Proteomics and Signal Transduction, Max Planck Institute of Biochemistry, Martinsried, Germany; ^3^Institute of Cardiology, Jagiellonian University Medical College, Krakow, Poland; ^4^Krakow Center for Medical Research and Technology, John Paul II Hospital, Krakow, Poland

**Supplemental Table 1.** Differentially changed proteins in the fibrin clots prepared *ex vivo* from plasma of patients with antiphospholipid syndrome (APS, n=23) as compared to healthy controls (n=20) and venous thromboembolism (VTE, n=19); additionally triple-positive APS was compared to double- and single-positive APS as well as APS was compared with regard to the presence or absence of lupus anticoagulant (LA) (p<0.05);
“-“ in the table indicates no significant differences between groups.

| **Protein ID** | **Protein names** | **Gene names** | **Fold change** | | | | | |
| --- | --- | --- | --- | --- | --- | --- | --- | --- |
|  |  |  | **APS vs. control** | **APS vs. VTE** | **VTE vs. control** | **Triple vs. Single** | **Triple vs. Double** | **LA vs. No LA** |
| P62258 | **14-3-3 protein epsilon** | YWHAE | 2.28 | - | - | - | - | - |
| P63104 | **14-3-3 protein zeta/delta** | YWHAZ | - | - | - | -3.65 | -4.42 | -3.92 |
| P63261 | **Actin, cytoplasmic 2** | ACTG1 | 2.17 | - | 1.89 | - | - | -2.38 |
| O15144 | **Actin-related protein 2/3 complex subunit 2** | ARPC2 | 2.73 | 1.79 | - | - | - | - |
| P59998 | **Actin-related protein 2/3 complex subunit 4** | ARPC4 | 3.3 | - | - | - | - | - |
| P61158 | **Actin-related protein 3** | ACTR3 | 3.31 | - | - | - | - | - |
| P04899 | **Adenylate cyclase-inhibiting G alpha protein** | GNAI2 | 6.44 | 2.51 | 2.57 | - | - | - |
| Q01518 | **Adenylyl cyclase-associated protein 1** | CAP1 | 2.91 | - | 2.21 | - | - | - |
| A0A024R6I7 | **Alpha-1-antitrypsin** | SERPINA1 | -1.37 | 3 | - | - | - | - |
| P08697 | **Alpha-2-antiplasmin** | SERPINF2 | -2.09 | - | - | - | - | - |
| P12814 | **Alpha-actinin-1** | ACTN1 | 3.17 | - | 3.72 | - | - | -6.12 |
| P06733 | **Alpha-enolase** | ENO1 | - | - | - | -5.43 | -11.29 | -7.15 |
| P01019 | **Angiotensinogen** | AGT | 2.7 | 2.79 | - | 5.78 | 3.8 | 4.94 |
| P07355 | **Annexin A2** | ANXA2 | - | - | -2.04 | - | - | - |
| P01008 | **Antithrombin-III** | SERPINC1 | -1.48 | -1.43 | - | - | - | -1.58 |
| P02647 | **Apolipoprotein A-I** | APOA1 | -1.5 | -1.25 | - | - | - | - |
| P06727 | **Apolipoprotein A-IV** | APOA4 | -1.68 | -1.6 | - | - | - | -1.84 |
| Q6Q788 | **Apolipoprotein A-V** | APOA5 | 2.51 | - | - | - | - | - |
| P04114 | **Apolipoprotein B-100** | APOB | 2.46 | 1.71 | - | - | - | - |
| P02655 | **Apolipoprotein C-II** | APOC2 | 1.89 | - | - | - | - | - |
| P02649 | **Apolipoprotein E** | APOE | 2.31 | 1.98 | - | - | - | - |
| O95445 | **Apolipoprotein M** | APOM | 2.32 | 2.44 | - | - | - | - |
| P08519 | **Apolipoprotein(a)** | LPA | 2.93 | - | - | - | - | - |
| Q06055-2 | **ATP synthase F(0) complex subunit C2, mitochondrial** | ATP5G2 | 2.56 | - | - | - | - | - |
| P25705 | **ATP synthase subunit alpha, mitochondrial** | ATP5A1 | 3.27 | - | 2.21 | - | - | - |
| P06576 | **ATP synthase subunit beta, mitochondrial** | ATP5B | 8.03 | - | 4.02 | - | - | - |
| P02749 | **Beta-2-glycoprotein 1** | APOH | 1.78 | - | - | - | - | - |
| P61769 | **Beta-2-microglobulin** | B2M | 4.72 | - | 3.16 | - | - | - |
| P13727 | **Bone marrow proteoglycan** | PRG2 | 38.87 | 23.43 | - | 35.68 | 23.34 | 30.43 |
| P12830 | **Cadherin-1** | CDH1 | -2.6 | - | - | - | - | -3.36 |
| P27824 | **Calnexin** | CANX | 2.81 | - | 2.17 | - | - | - |
| P15169 | **Carboxypeptidase N catalytic chain** | CPN1 | -1.4 | - | - | - | - | - |
| O43866 | **CD5 antigen-like** | CD5L | - | 1.7 | - | - | - | - |
| Q00610 | **Clathrin heavy chain 1** | CLTC | 4.02 | - | - | - | - | - |
| P00488 | **Coagulation factor XIII A chain** | F13A1 | -1.57 | -1.49 | - | - | - | -1.59 |
| P23528 | **Cofilin-1** | CFL1 | - | - | 3.12 | -10.54 | -15.0 | -12.03 |
| P02745 | **Complement C1q subcomponent subunit A** | C1QA | - | 2.62 | - | - | - | - |
| P02747 | **Complement C1q subcomponent subunit C** | C1QC | - | 1.99 | - | - | - | - |
| P01024 | **Complement C3** | C3 | - | - | - | - | - | 1.39 |
| P0C0L4 | **Complement C4-A** | C4A | -1.55 | - | -1.87 | - | - | - |
| P0C0L5 | **Complement C4-B** | C4B | - | - | - | - | - | 1.61 |
| P01031 | **Complement C5** | C5 | 1.58 | 1.6 | - | 1.85 | 2.29 | 2.0 |
| P13671 | **Complement component C6** | C6 | 3.52 | 2.83 | - | 3.16 | 2.64 | 2.95 |
| P10643 | **Complement component C7** | C7 | 5.57 | 4.15 | - | 3.36 | 3.78 | 3.51 |
| P07357 | **Complement component C8 alpha chain** | C8A | 3.18 | 2.46 | - | 2.76 | 3.26 | 2.94 |
| P07358 | **Complement component C8 beta chain** | C8B | 3.36 | 2.81 | - | 2.55 | 2.51 | 2.53 |
| P07360 | **Complement component C8 gamma chain** | C8G | 4.03 | 2.96 | - | 2.78 | 8.18 | 4.17 |
| P02748 | **Complement component C9** | C9 | 3.28 | 2.21 | - | 3.37 | 3.75 | 3.51 |
| P00403 | **Cytochrome c oxidase subunit 2** | MT-CO2 | 2.15 | - | - | - | - | - |
| O00429 | **Dynamin-1-like protein** | DNM1L | 2.95 | - | - | - | - | - |
| Q12805 | **EGF-containing fibulin-like extracellular matrix prot 1** | EFEMP1 | - | - | - | -2.69 | -2.42 | -2.59 |
| Q9Y6C2 | **EMILIN-1** | EMILIN1 | 2.18 | - | 1.98 | - | - | - |
| O94919 | **Endonuclease domain-containing 1 protein** | ENDOD1 | 2.86 | - | - | - | - | - |
| P14625 | **Endoplasmin** | HSP90B1 | 3.19 | 2.56 | - | - | - | - |
| P27105 | **Erythrocyte band 7 integral membrane protein** | STOM | 4.12 | - | 2.85 | - | - | - |
| Q16610 | **Extracellular matrix protein 1** | ECM1 | -1.5 | -1.4 | - | - | - | - |
| Q86UX7 | **Fermitin family homolog 3** | FERMT3 | 2.38 | - | 2.93 | -3.86 | -4.46 | -4.07 |
| Q9UGM5 | **Fetuin-B** | FETUB | -2.44 | -2.18 | - | -2.94 | -3.06 | -2.99 |
| Q08830 | **Fibrinogen-like protein 1** | FGL1 | -2.67 | -2.25 | - | - | - | -4.92 |
| P02751-8 | **Fibronectin** | FN1 | -1.79 | -1.67 | - | - | - | - |
| O75636 | **Ficolin-3** | FCN3 | - | 1.8 | - | - | - | - |
| P21333-2 | **Filamin-A** | FLNA | 3.02 | - | 2.91 | - | - | -2.5 |
| P06396 | **Gelsolin** | GSN | - | - | 1.33 | - | - | - |
| Q14789-2 | **Golgin subfamily B member 1** | GOLGB1 | -2.49 | -2.52 | - | - | - | - |
| P62879 | **G protein subunit beta-2** | GNB2 | 2.78 | - | - | - | - | - |
| A0A0G2JIW1 | **Heat shock 70 kDa protein 1B** | HSPA1B | - | - | - | -3.29 | -10.44 | -5.08 |
| P11142 | **Heat shock cognate 71 kDa protein** | HSPA8 | - | - | - | -5.65 | -12.32 | -7.57 |
| P26927 | **Hepatocyte growth factor-like protein** | MST1 | -1.52 | -1.7 | - | - | - | - |
| P04196 | **Histidine-rich glycoprotein** | HRG | -1.38 | -1.37 | - | - | - | -1.59 |
| Q6FI13 | **Histone H2A type 2-A** | HIST2H2AA3 | - | 4.55 | - | - | - | - |
| Q99880 | **Histone H2B type 1-L** | HIST1H2BL | - | 3.44 | - | - | - | - |
| P30498 | **HLA class I histocompatibility antigen** | HLA-B | 3.51 | 2.58 | - | - | - | - |
| P01765 | **Ig heavy chain V-III region TIL** | IGHV3-23 | - | 1.85 | - | - | - | - |
| P01600 | **Ig kappa chain V-I region Hau** | IGKV1-39 | 1.87 | 1.89 | - | - | - | - |
| P18135 | **Ig kappa chain V-III region HAH** | IGKV3-20 | - | 2.09 | - | - | - | - |
| P01623 | **Ig kappa chain V-III region WOL** | IGKV3-20 | - | 2.36 | - | - | - | - |
| A0A087WYJ9 | **Ig mu chain C region** | IGHM | 1.83 | 1.91 | - | - | - | - |
| P01591 | **Immunoglobulin J chain** | IGJ | 1.94 | 2.55 | - | - | - | - |
| A0A087WWC9 | **Immunoglobulin lambda-like polypeptide 5** | IGLL5 | - | 1.32 | - | - | - | - |
| A0A075B6I2 | **Immunoglobulin lambda variable 6-57** | IGLV6-57 | 3.56 | 4.84 | - | - | - | - |
| A0A075B6J4 | **Immunoglobulin lambda variable 3-25** | IGLV3-25 | 3.6 | 3.2 | - | - | - | - |
| A0A075B6J8 | **Immunoglobulin lambda variable 3-19** | IGLV3-19 | 2.72 | 2.92 | - | - | - | - |
| A0A087WW89 | **Immunoglobulin heavy variable 3-72** | IGHV3-72 | 1.58 | 1.78 | - | - | - | - |
| A0A0G2JN58 | **Immunoglobulin heavy variable 4-59** | IGHV4-39 | 2.61 | 1.98 | - | - | - | - |
| P08514 | **Integrin alpha-IIb** | ITGA2B | 6.37 | - | 4.47 | - | - | - |
| P05556 | **Integrin beta-1** | ITGB1 | 3.61 | - | 2.84 | - | - | - |
| P05106 | **Integrin beta-3** | ITGB3 | 5.72 | - | 4.32 | - | - | - |
| Q13418 | **Integrin-linked protein kinase** | ILK | 4.46 | - | 4.2 | -3.69 | -4.28 | -3.90 |
| P19823 | **Inter-alpha-trypsin inhibitor heavy chain H2** | ITIH2 | -1.34 | -1.28 | - | - | - | - |
| Q14624 | **Inter-alpha-trypsin inhibitor heavy chain H4** | ITIH4 | - | -1.28 | - | -1.69 | -1.67 | -1.68 |
| P48735 | **Isocitrate dehydrogenase [NADP], mitochondrial** | IDH2 | 2.84 | - | 2.05 | - | - | - |
| P29622 | **Kallistatin** | SERPINA4 | 2.7 | 2.39 | - | 4.32 | 3.02 | 3.78 |
| P01042 | **Kininogen-1** | KNG1 | - | -1.27 | - | - | - | - |
| P07195 | **L-lactate dehydrogenase B chain** | LDHB | - | - | - | -1.97 | - | -3.02 |
| P18428 | **Lipopolysaccharide-binding protein** | LBP | -1.61 | -1.43 | - | - | - | - |
| P61626 | **Lysozyme C** | LYZ | - | - | -2.39 | - | - | - |
| Q13201 | **Multimerin-1** | MMRN1 | 6.79 | - | 4.95 | - | - | -2.08 |
| P05164 | **Myeloperoxidase** | MPO | 2.37 | - | - | - | - | - |
| P60660 | **Myosin light polypeptide 6** | MYL6 | 5.16 | - | 3.39 | - | - | - |
| P19105 | **Myosin regulatory light chain 12A** | MYL12A | 9.08 | - | 3.69 | - | - | - |
| P35579 | **Myosin-9** | MYH9 | 4.11 | 1.85 | 2.23 | - | - | - |
| P00387 | **NADH-cytochrome b5 reductase 3** | CYB5R3 | 4.96 | - | 3.24 | - | - | - |
| P14543 | **Nidogen-1** | NID1 | - | - | - | - | - | -1.89 |
| P62937 | **Peptidyl-prolyl cis-trans isomerase A** | PPIA | - | - | - | -12.51 | -22.02 | -15.46 |
| P51659 | **Peroxisomal multifunctional enzyme type 2** | HSD17B4 | 2.48 | 2.57 | - | - | - | - |
| P03952 | **Plasma kallikrein** | KLKB1 | - | - | - | -1.4 | - | -1.61 |
| P16671 | **Platelet glycoprotein 4** | CD36 | 5.62 | 2.59 | - | - | - | - |
| P07359 | **Platelet glycoprotein Ib alpha chain;Glycocalicin** | GP1BA | 5.98 | - | 6.04 | - | - | - |
| P13224 | **Platelet glycoprotein Ib beta chain** | GP1BB | 6.09 | - | 4.55 | - | - | - |
| P14770 | **Platelet glycoprotein IX** | GP9 | 6.36 | - | 3.4 | - | - | - |
| P08567 | **Pleckstrin** | PLEK | - | - | 2.59 | -7.06 | -5.69 | -6.51 |
| P07737 | **Profilin-1** | PFN1 | - | -2.94 | - | -14.07 | -17.96 | -15.42 |
| P23219 | **Prostaglandin G/H synthase 1** | PTGS1 | 5.13 | - | - | - | - | - |
| P30101 | **Protein disulfide-isomerase A3** | PDIA3 | - | - | - | -4.53 | -5.58 | -4.90 |
| Q92954-3 | **Proteoglycan 4** | PRG4 | - | -2.13 | - | - | - | - |
| P00734 | **Prothrombin** | F2 | -2.02 | -1.79 | - | - | - | - |
| Q15404 | **Ras suppressor protein 1** | RSU1 | 2.17 | - | 2.55 | -3.92 | -4.66 | -4.18 |
| Q9H0U4 | **Ras-related protein Rab-1B** | RAB1B | 3.42 | - | 2.75 | - | - | - |
| O00194 | **Ras-related protein Rab-27B** | RAB27B | 5.42 | - | 2.69 | - | - | - |
| P61224 | **Ras-related protein Rap-1b** | RAP1B | - | -2.19 | 3.55 | -5.03 | -6.38 | -5.50 |
| Q9NQC3 | **Reticulon-4;Reticulon** | RTN4 | 3.54 | - | - | - | - | - |
| Q99969 | **Retinoic acid receptor responder protein 2** | RARRES2 | -2.27 | - | - | - | - | - |
| P04844 | **Ribophorin-2** | RPN2 | 1.73 | 1.97 | - | - | - | - |
| P16615 | **Sarcoplasmic reticulum calcium ATPase 2** | ATP2A2 | 2.19 | - | - | - | - | - |
| Q93084-2 | **Sarcoplasmic reticulum calcium ATPase 3** | ATP2A3 | 3.32 | - | - | - | - | - |
| Q13103 | **Secreted phosphoprotein 24** | SPP2 | 2.08 | 2.12 | - | - | - | - |
| P49908 | **Selenoprotein P** | SEPP1 | -1.57 | - | - | - | - | - |
| P35542 | **Serum amyloid A-4 protein** | SAA4 | 1.8 | 2.26 | - | - | - | - |
| O95810 | **Serum deprivation-response protein** | SDPR | 8.05 | - | 3.97 | - | - | - |
| P11169 | **Solute carrier family 2** | SLC2A3 | 6.37 | - | 4.15 | - | - | - |
| Q9Y490 | **Talin-1** | TLN1 | 3.48 | - | 2.38 | - | - | - |
| P07996 | **Thrombospondin-1** | THBS1 | 3.33 | - | 2.48 | - | - | - |
| P24557 | **Thromboxane-A synthase** | TBXAS1 | 5.03 | 2.59 | 1.95 | - | - | - |
| P01137 | **Transforming growth factor beta-1** | TGFB1 | 2.84 | - | 2.21 | - | - | - |
| P55072 | **Transitional endoplasmic reticulum ATPase** | VCP | 2.98 | - | - | - | - | - |
| Q86YW5 | **Trem-like transcript 1 protein** | TREML1 | 4.98 | - | 3.19 | - | - | - |
| P06753-4 | **Tropomyosin alpha-3 chain** | TPM3 | 2.46 | - | 2.59 | -4.46 | -5.65 | -4.87 |
| P68363 | **Tubulin alpha-1B chain** | TUBA1B | 3.4 | - | 2.63 | - | - | - |
| P68366 | **Tubulin alpha-4A chain** | TUBA4A | 7.57 | - | 4.29 | - | - | - |
| Q9H4B7 | **Tubulin beta-1 chain** | TUBB1 | 7.66 | - | 4.65 | - | - | - |
| P68371 | **Tubulin beta-4B chain** | TUBB4B | 4.38 | - | 2.29 | - | - | - |
| P18206 | **Vinculin** | VCL | 6.06 | - | 3.7 | - | - | - |
| D6RF35 | **Vitamin D-binding protein** | GC | 2.41 | 2.57 | - | 4.57 | 3.14 | 3.97 |
